# A perspective on oligonucleotide therapy: Approaches to patient customization

**DOI:** 10.3389/fphar.2022.1006304

**Published:** 2022-10-19

**Authors:** Shikha Thakur, Apurba Sinhari, Priti Jain, Hemant R. Jadhav

**Affiliations:** ^1^ Pharmaceutical Chemistry Laboratory, Department of Pharmacy, Birla Institute of Technology and Sciences Pilani, Pilani, RJ, India; ^2^ Department of Pharmaceutical Chemistry, School of Pharmaceutical Sciences, Delhi Pharmaceutical Sciences and Research University, New Delhi, India

**Keywords:** oligonucleotide therapeutics, antisense oligonucleotide, antisense RNA, microRNA, bioconjugation

## Abstract

It is estimated that the human genome encodes 15% of proteins that are considered to be disease-modifying. Only 2% of these proteins possess a druggable site that the approved clinical candidates target. Due to this disparity, there is an immense need to develop therapeutics that may better mitigate the disease or disorders aroused by non-druggable and druggable proteins or enzymes. The recent surge in approved oligonucleotide therapeutics (OT) indicates the imminent potential of these therapies. Oligonucleotide-based therapeutics are of intermediate size with much-improved selectivity towards the target and fewer off-target effects than small molecules. The OTs include Antisense RNAs, MicroRNA (MIR), small interfering RNA (siRNA), and aptamers, which are currently being explored for their use in neurodegenerative disorders, cancer, and even orphan diseases. The present review is a congregated effort to present the past and present of OTs and the current efforts to make OTs for plausible future therapeutics. The review provides updated literature on the challenges and bottlenecks of OT and recent advancements in OT drug delivery. Further, this review deliberates on a newly emerging approach to personalized treatment for patients with rare and fatal diseases with OT.

## 1 Introduction

Oligonucleotide (ON) and oligonucleotide therapeutics (OT) can potentially treat a diverse range of diseases that are often associated with undruggable proteins. ON are small single or double-stranded segments of DNA or RNA molecules made up of 10–50 nucleotides. These have been extensively applied to molecular diagnostics, genetic testing, microarrays, gene therapy, forensics, and research (Lundin et al., 2015). Most oligonucleotides (ON) bind to their targets using complementary Watson-Crick base pairing rules (Khvorova and Watts, 2017). OTs usually act *via* preventing, replacing, adding, or editing RNA and DNA at the molecular level. In contrast to small conventional molecules that require much larger optimization, ON modalities can be designed based on the primary sequence knowledge. The challenge in small molecules is off-target toxicity and undruggable protein targets, whereas ON allows for precision and personalized treatment with minimal off-target effects (Al, 2002; Overington et al., 2006; Dixon and Stockwell, 2009; Roberts et al., 2020). The success of ON is defined by its ability to affect the target and its pharmacokinetic parameters. ON has emerged as a new therapeutic platform that has made remarkable progress (Cavagnari, 2011). The recent interest in these therapeutics is attributed to their potential to treat undruggable targets and to better pharmacokinetics parameters that include distribution to major body compartments excluding the brain. Earlier, administration was considered the drawback for most OTs due to poor absorption *via* the oral route. However, research is now piling up that discloses the enhancement of oral administration and intestinal permeability upon the concomitant use of absorption enhancers (for example, sodium caprate). They are metabolized by exo- and endo-nucleases in contrast to the cytochrome P_450_ enzyme systems utilized by the small molecules. The excretion by the liver or kidney follows the metabolism. Another added advantage of using OT is their minimal side effects. OTs target the replicating DNA or RNA and hence are highly selective for their marked sequence, unlike small molecules that show a plethora of off-target effects. The therapeutic nucleic acids bind to complementary nucleic acids in accordance with the Watson-Crick base pairing models, thereby allowing rapid and prudent development of drugs against genetic targets.

**FIGURE 1:**
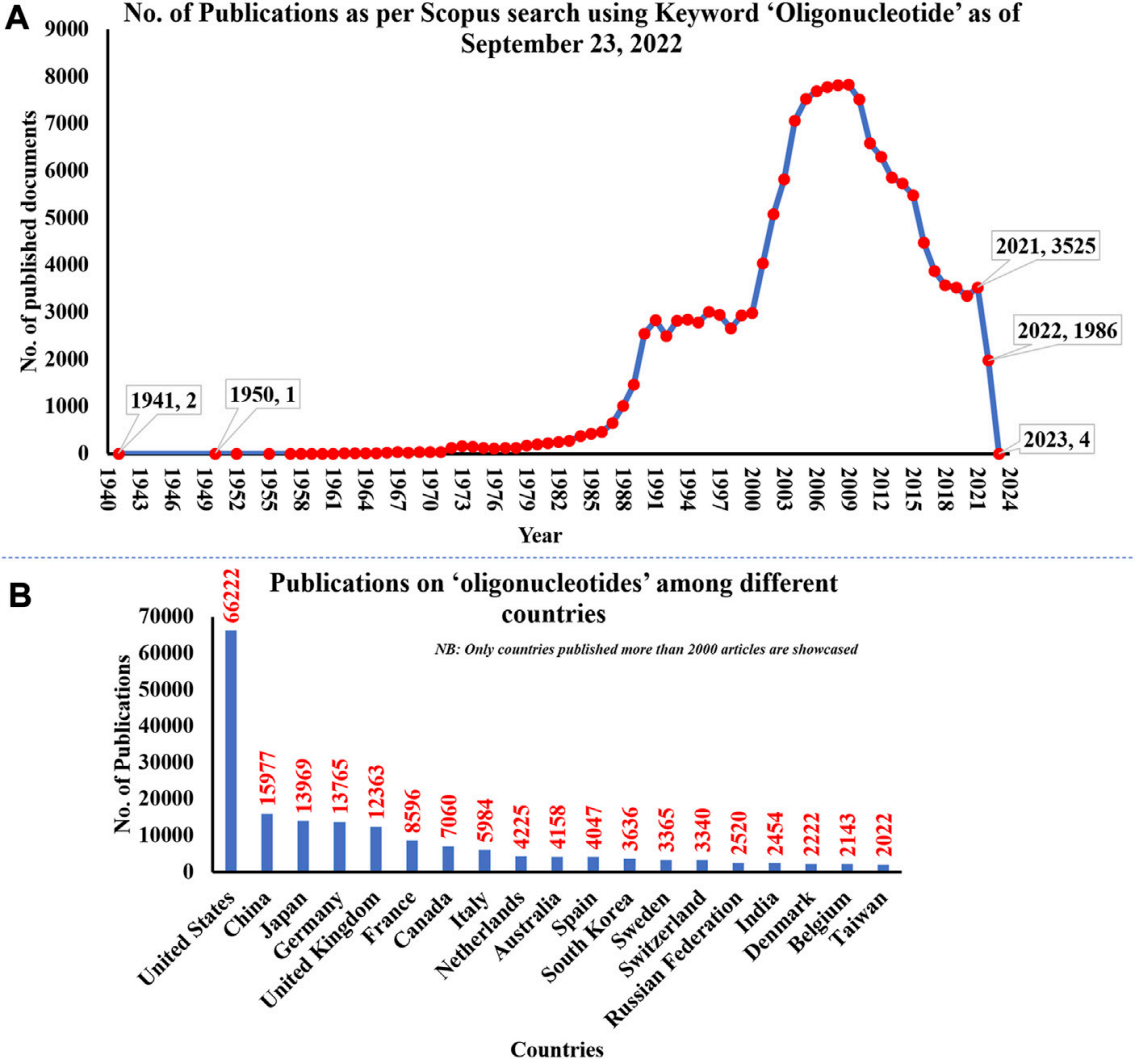
**(A)** The number of publications on oligonucleotides till 2022; **(B)** Publication date from major countries. The data was obtained from the Scopus database on 23 September 2022.

**FIGURE 2:**
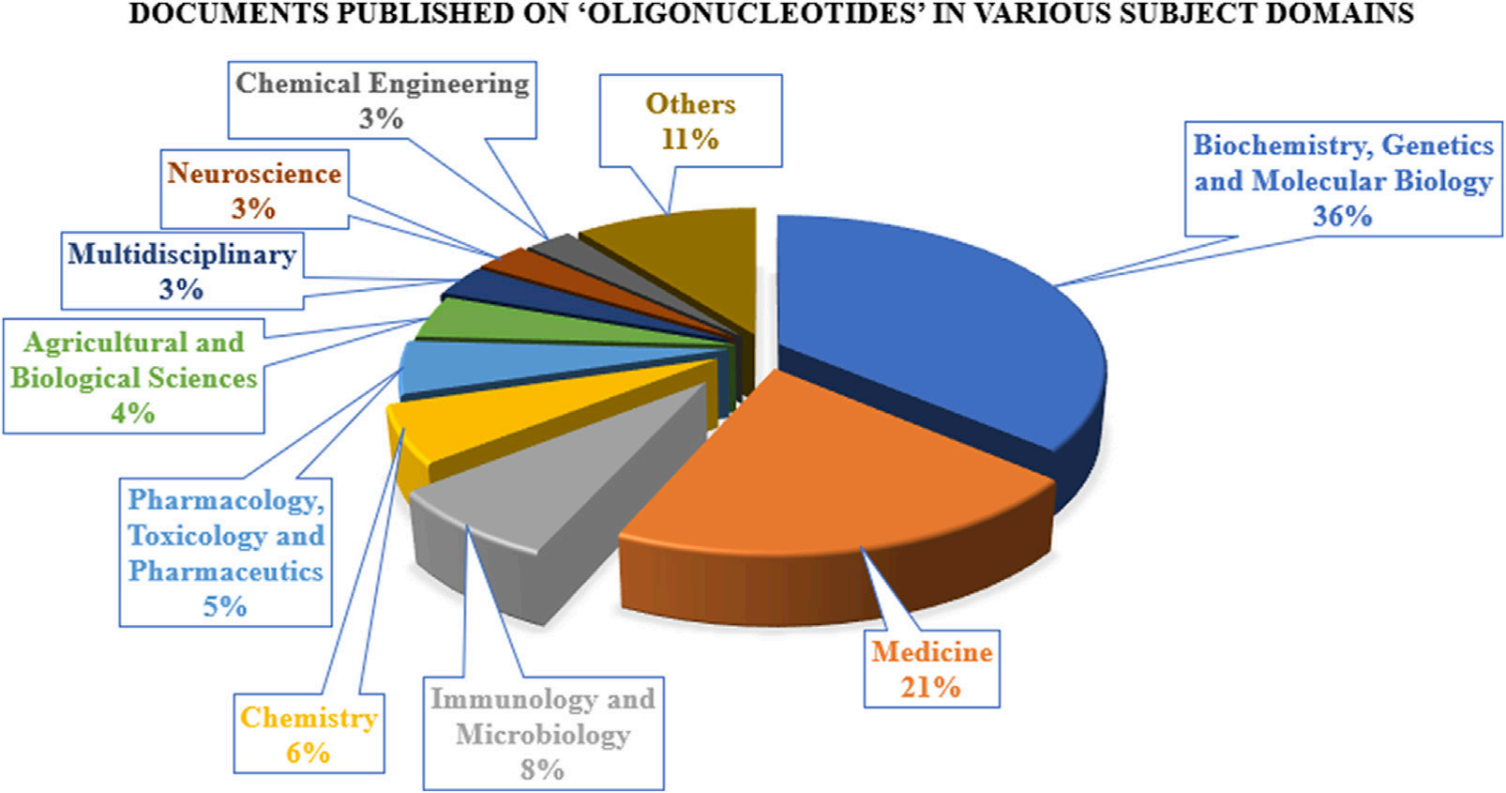
Documents published on “oligonucleotides” in various subject domains.

**FIGURE 3:**
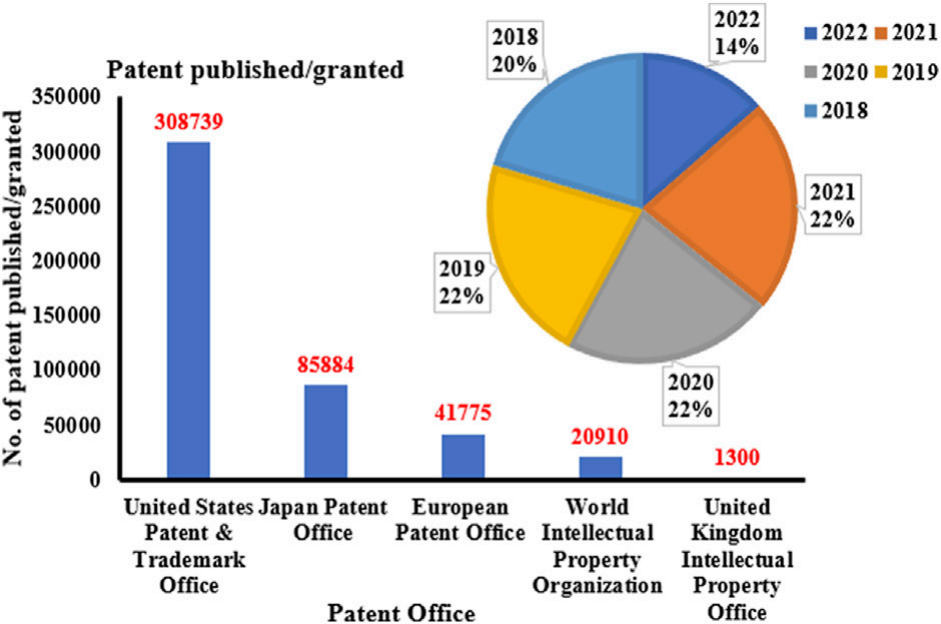
Patents published/granted on oligonucleotide by different agencies in the last 5 years.

The research on ON and associated therapies is increasing at a tremendous pace. A quick search made using the Scopus database (last accessed on 23 September 2022) on the keyword “oligonucleotide” revealed a total of 160,064 documents published, among which 137,312 documents have published as research-based articles, with the first article coming in 1941 (Figure 1A). Figure 1B indicates publication data from major countries of the world. The majority of the published work (Figure 2) is in the broader area of biochemistry or molecular biology (36%), followed by medicine (21%), immunology (8%), chemistry (6%), and medicine (5%) showing their significance in biological field for the use in the therapeutic or diagnostic agent development. Apart from journal publications, several patents granted by different patent offices, such as the United States Patent and Trademarks Office (USPTO), Japan Patent Office (JPO), etc., are given in Figure 3. It is estimated that by 2024, the global OT market will be approximately US $7 billion (Wraight and White, 2001). Currently, 15 ONs are in the clinical arena, in addition to several clinical trials for neurological, cardiovascular, hepatic, and ophthalmic disorders. Considering this topic’s attention, this review attempts to collate the available information on oligonucleotide-based drug therapy and RNA-based therapeutics, as well as focus on challenges faced by oligonucleotides and recent advances in improving oligonucleotide drug delivery. Further, this review deliberates on a newly emerging approach to personalized treatment for patients with rare and fatal diseases with OT.

## 2 Historical developments of oligonucleotide therapeutics

In the early 1960s, ON synthesis started with phosphorothioate backbone modification (replacement of oxygen atom in backbone of the phosphate group backbone with a sulfur atom) that increased nuclease resistance of the nucleic acid, which led to greater protein-binding (Gait and Sheppard, 1976; Khorkova and Wahlestedt, 2017). In 1977, the expression of a gene manipulated using exogenous nucleic acids by inhibiting complementary RNA translation by using single-stranded DNA was reported (Paterson et al., 1977; Oberemok et al., 2018). Zamecnik and Stephenson further pioneered the notion of antisense oligonucleotides (ASO) in 1978 when they found a 13-m-long oligodeoxynucleotide inhibiting the Rous sarcoma virus proliferation and cell transformation in the chicken embryo (Zamecnik and Stephenson, 1978).

**FIGURE 4:**
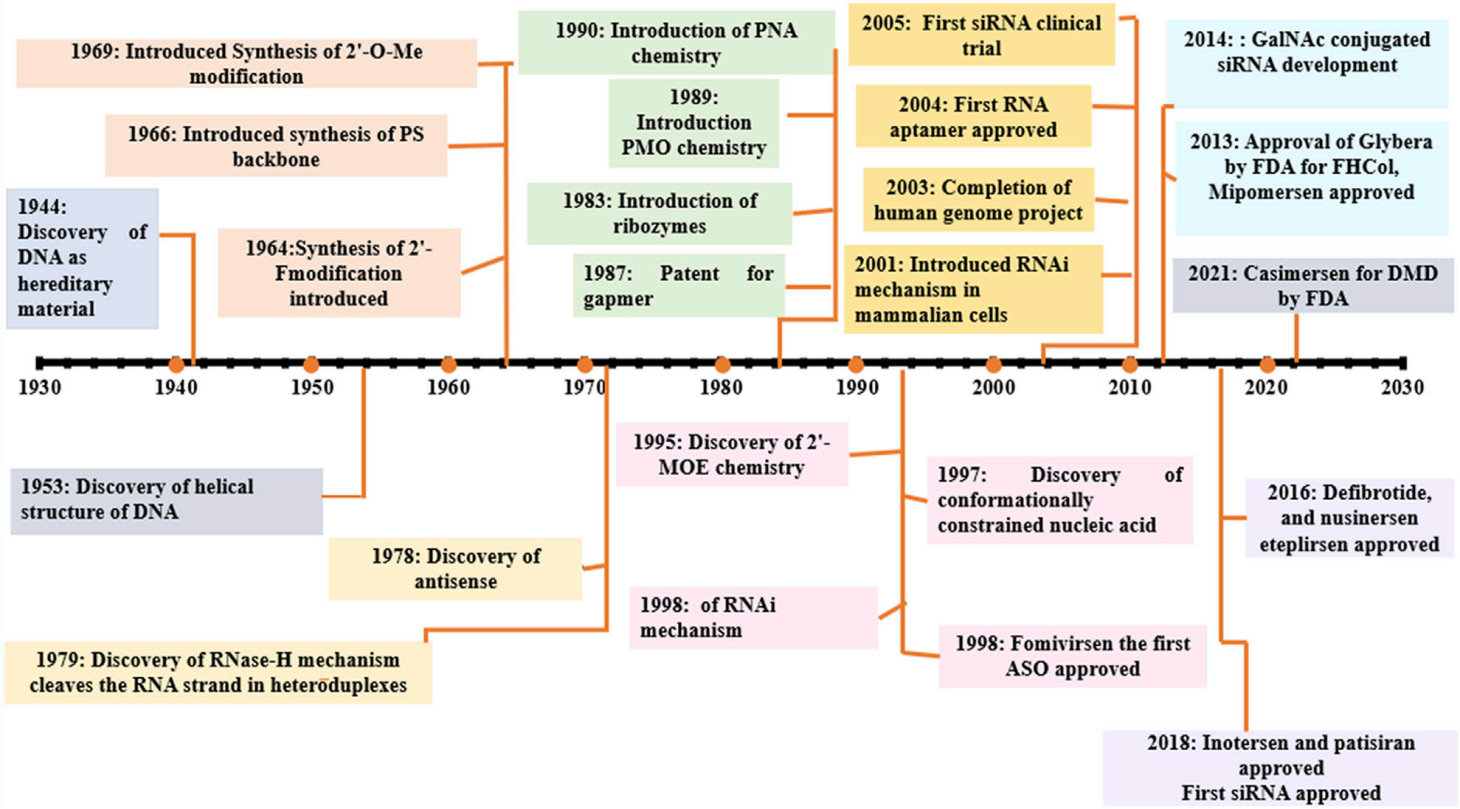
Selected milestones in the historical development of OT; 2′-O-Me: 2′-O-methylation, 2′F: 2′ fluoro, PS, phosphorothioated; PNA, peptide nucleic acid; PMO, phosporodiamidate morpholino; ASO, antisense oligonucleotide; GAPMER, short DNA ASO with RNA segments; FHCol, familial hyperchloestromia; GalNAc, N-acetylgalactosamine.

Donis and Keller discovered in 1979 that RNase H recognizes and cuts the RNA strand in RNA-DNA heteroduplexes, and catalyzes the destruction of immature pre-mRNAs, proving that ASOs degrade target RNA through an enzyme-mediated mechanism (Donis-Keller, 1979). Matsukura et al. further promoted the growth of ASOs for various clinical applications. In 1989, the concept of aptamers as a type of oligonucleotide for treating HIV-1 was first described. Further, in the 1990s, the idea of miRNAs, known as microRNAs, was described. Fomivirsen, the first antisense medicine with a modified phosphodiester backbone having 21-oligonucleotides with phosphorothioate linker, was licensed by the US FDA (United States Food and Drug Administration) in 1998 for the treatment of cytomegalovirus (CMV) retinitis in AIDS patients (Roehr, 1998; de Smet et al., 1999). Also, in 1998, Fire et al. found RNA interference (RNAi) as a revolutionary biological process in *Caenorhabditis elegans* (Fire et al., 1998; Timmons, 2006). The RNAi phenomenon was first reported in mammalian cells in 2001. Since then, the RNAi method has been utilized to develop several novel medications, some of which are currently in clinical trials. In 2004, the US FDA approved pegaptanib, a second RNA-based drug. After the discovery of RNAi, the first clinical trial of siRNA was reported in 2005. In 2018, the first siRNA drug, “patisiran” was approved by USFDA. The field of ON therapeutics has been expanding swiftly for the past 20 years. Currently, ON-based therapeutics are gaining widespread recognition for their use against various human diseases like Huntington’s disease (Sah and Aronin, 2011), Alzheimer’s disease (Hinrich et al., 2016), Duchenne muscular dystrophy (DMD) (Lu et al., 2011; Lim et al., 2017), and respiratory illnesses such as coronary obstructive pulmonary diseases (COPD), asthma, and lung cancer (Séguin and Ferrari, 2009; Crooke, 2017; Mehta et al., 2019). The timeline of key discoveries of OTs is shown in Figure 4. Till 2022, fifteen drugs based on ON therapy received regulatory approval from US FDA, as indicated in Table 1. There are over 246 studies registered for clinical trials and of which 22 studies are Phase 3 trials (Table 2).

**TABLE 1:** Oligonucleotides approved by the US FDA.

Generic name (brand name)	Indication	Year of approval	Target organ and administration	Mechanism of action	Chemical class	Drug bank ID and marketing status
Fomivirsen (Vitravene)	Cytomegalovirus retinitis (Antiviral)	August 1998 (Ionis Pharma, Novartis)	Eye, intravitreal injection	Inhibits the replication of human CMV	ASO	DB06759 (Discontinued)
Pegaptanib (Macugen)	Neovascular age-related muscular degeneration	September 2004 (Pfizer/Eyetech)	Eye, intravitreal	Inhibits Vascular endothelial growth factor A	Aptamer	DB045895 (Discontinued)
Mipomersen (Kynamro)	Homozygous familial hypercholesterolemia	January 2013 (Sanofi/Isis)	Liver, subcutaneous	Hamper translation of ApoB-100 mRNA	ASO	DB05528 (Discontinued)
Defibrotide (Defitelio)	Severe hepatic veno-occlusive disease, Sinusoidal Obstruction Syndrome (SOS)	30 March 2016 (Jazz Pharma)	Liver, subcutaneous	Inhibitor of plasminogen activator	ON mixture	DB04932 (Available)
Eteplirsen (Exondys 51)	Duchenne muscular dystrophy (DMD)	September 2016 (Sarepta)	Hand, intravenous	Alteration in exon splicing by binding to dystrophin	Morpholino	DB06014 (Available)
Nusinersen (Spinraza)	Spinal muscular atrophy	December 2016 (Biogen/Ionis)	CNS, intrathecal	Inhibits the SMN2 pre-RMA splicing	ASO	DB13161 (Available)
Inotersen (Tegsedi)	Hereditary transthyretin-mediated amyloidosis	October 2018 (Ionis)	Liver, subcutaneous	Degradation of wild-type and mutant TTR mRNA	ASO	DB14713 (Available)
Patisiran (Onpattro)	Polyneuropathy of hereditary transthyretin-mediated amyloidosis (hATTR)	August 2018 (Alnylam Pharmaceuticals)	Liver, subcutaneous	Degradation of wild-type and mutant Transthyretin mRNA	siRNA	DB14582 (Available)
Volanesorsen (Waylivra)	Familial Chylomicronaemia	2019 (Ionis)	Liver, subcutaneous	Inhibits apolipoprotein C-III	ASO	DB15067 (Available)
Givosiran (Givlaari)	Acute hepatic porphyria	November 2019 (Alnylam)	Hepatic, subcutaneous	Inhibits hepatic aminolevulinic acid synthase (ALAS1) mRNA	siRNA	DB14901 (Available)
Golodirsen (Vyondis 53)	DMD	December 2019 (Sarepta)	Hand, intravenous	Induce exon splicing	Morpholino	DB15593 (Available)
Viltolarsen (Viltepso)	DMD	August 2020 (NS Pharma)	Hand, intravenous	Exclude the exon *via* binding to exon 53 of dystrophin pre-mRNA	Morpholino	DB15005 (Available)
Lumasiran (Oxlumo)	Hyperoxaluria type 1	November 2020 (Alnylam)	Liver, subcutaneous	Inhibits the translation of enzyme glycolate oxidase (GO) or Hydroxy acid oxidase 1 (HAO1)	siRNA	DB15935 (Available)
Inclisiran (Leqvio)	Primary hypercholesteremia (heterozygous familial and non-familial)	December 2021 (Novartis/Alnylam)	Liver, subcutaneous	Prevent the hepatic translation proprotein convertase subtilisin-Kexin type 9 (PCSK9)	siRNA	DB14901 (Available)
Casimersen (Amondys-45)	DMD	February 2021 (Sarepta therapeutics)	Hand, intravenous	Binds to exon 45 of the DMD pre-mRNA prevent translation	ASO	DB14984 (Available)

**TABLE 2:** Oligonucleotides in Phase 3 clinical trials (http//www.ClinicalTrials.gov).

Disease area	Compound	Indication	Target	Chemical class	NCT ID
Neurological	Tominersen	Huntingtons’ disease	Huntingtin protein (HTT)	ASO	NCT03761849
	Tofersen	Amyotrophic lateral sclerosis (ALS)	Superoxide dismutase type-1 (SOD1)	ASO	NCT02623699
Cardiovascular	Pelacarsen	Elevated lipoprotein (Lp)	Apolipoprotein(a)	ASO	NCT04023552
	AMG 890	Elevated lipoprotein (Lp)	Apolipoprotein(a)	siRNA	NCT03626662
	AKCEA– transthyretin (TTR)-LRX	TTR amyloidosis	Transthyretin (TTTR) protein	ASO	NCT04136184
	Vutrisiran	TTR amyloidosis	TTR protein	siRNA	NCT03759379
	CGT003	Cornory artery diseases	Transcription factor E2F	Decoy	NCT00041925, NCT00042081
Hepatic	Nidosiran	Primary hyperoxaluria type 1, 2 and 3	Lactate dehydrogenase (LDHA)	siRNA	NCT04042402
	JNJ-3989	Hepatitis B (HBV)	RNAi	siRNA	NCT04129554
Hematologic	Fitusiran	Hemophilia and rare bleeding disorder (RBD)	Antithrombin	siRNA	NCT03417245
	Cemdisiran	Peroxysmal Nocturnal Hemoglobinuria	C5	ASO	NCT05070858, NCT05133531, NCT05131204
Metabolic	Alicaforsen	Crohn’s disease	Intracellular adhesion molecules (ICAM-1)	ASO	NCT00048295
Ophthalmic	Sepofarsen	Leber congenital amaurosis 10	CEP290 gene, p. Cys998X mutation	ASO	NCT03913143
	QR-421a	Retinitis pigmentosa	Usher syndrome (USH2A gene)	ASO	NCT05176717
		Dry eye disease			
	Ultevursen	Retinitis pigmentosa	USH2A gene	ASO	NCT05158296
	Tivanisiran	Sjögren’s Syndrome	Transient Receptor Potential Vanilloid 1 (TRPV-1)	siRNA	NCT04819269,NCT03108664
	Cosdosiran (QPI-1007)	Caspase-2	Non-arteritic anterior ischemic optic neuropathy (NAION)	siRNA	NCT01965106
Dermatological	Tilsotolimod	Anti-programmed cell death protein 1 (PD-1) refractory metastatic melanoma	Toll-like receptor 9 (TLR9)	ASO	NCT03865082
Renal	Teprasiran (QPI-1002)	Acute kidney injury	p53	siRNA	NCT02610296, NCT03510897
Cancer	Oblimersen	Melanoma	Bcl-2	ASO	NCT00016263, NCT00543205, NCT00030641

## 3 Types of oligonucleotides and their mechanism of action

Oligonucleotide-based therapeutics can act through various mechanisms depending on their structure and chemistry. The therapeutic nucleic acids bind to complementary nucleic acids by Watson-Crick base pairing. Based on their mechanism of action, oligonucleotides are classified into the following: Antisense oligonucleotide (ASO), RNA interference (RNAi) such as small interfering RNA (siRNA), microRNA (miRNA), CpG oligonucleotide, Aptamers, Clustered regularly interspaced palindromic repeats **(**CRISPR), and decoys (Kim, 2020).

### 3.1 Antisense oligonucleotides

**FIGURE 5:**
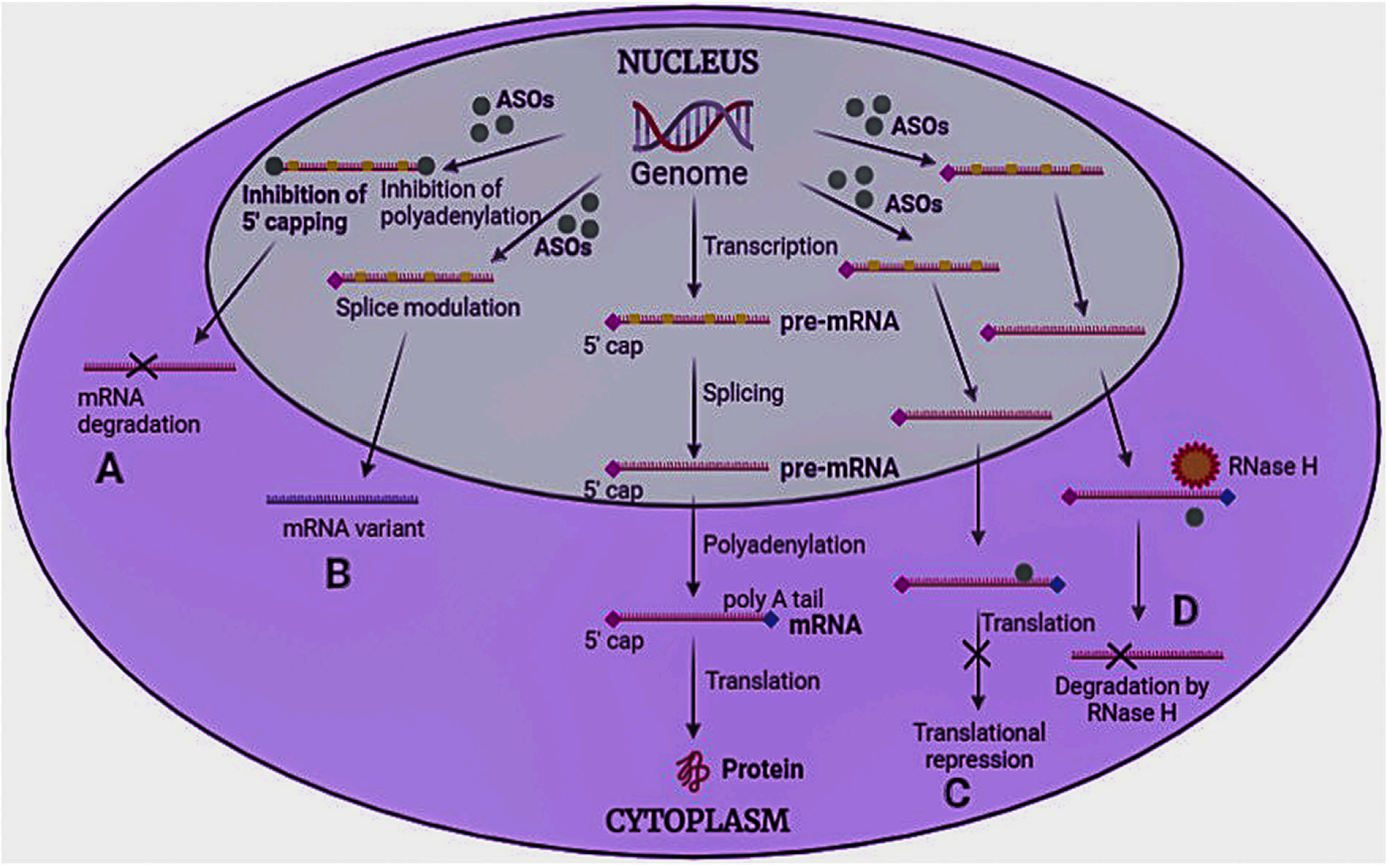
Mechanism of antisense oligonucleotides. ASOs bind to pre-mRNA/mRNA and act in four ways to inhibit protein synthesis **(A)** Inhibition of 5′ capping and polyadenylation of the tail, **(B)** Modulation of RNA splicing, **(C)** Translational repression, and **(D)** RNase H induced degradation.

ASOs are oligodeoxyribonucleotides representing a systemic and specific gene silencing strategy (Lennox and Behlke, 2016). These are small, single-stranded (8–50 bases), and can modify RNA and impede protein expression. In 1998, US FDA approved the first antisense oligonucleotide “Fomivirsen” (Perry and Balfour, 1999). ASOs work through numerous mechanisms (Figure 5), broadly divided into two groups 1) splicing modulator and steric-blocker oligonucleotides, and 2) gene-expression inhibitors (RNase H induced degradation and RNA interference).

Splicing modulators prevent the process of splicing in translation. These generally target specific codons, such as the AUG initiation codon. Splicing can be of two types: a) Exon skipping and b) Exon inclusions. In exon skipping, ASOs attach to pre-mRNA splicing machinery to skip the exons that carry mutations, repair the faulty RNA frame, and functional proteins to be produced (Rinaldi and Wood, 2018). However, in exon inclusion, ASOs attach to pre-mRNA and prevent splicing machinery from accessing the transcript sites (Kole et al., 2012). Splice-modulating oligonucleotides may be helpful in treating disorders with splicing defects or where the target exons contain disease-causing mutations. Since more than 95% of all human genes produce splice variant proteins, modulation of splicing machinery may be beneficial in many diseases. Steric blocker OTs induce steric hindrance by binding to the target RNA sequence and cause translational arrest by inhibiting ribosomal activity.

Gene expression inhibitors cause the selective breakdown of DNA-RNA heteroduplexes *via* RNase enzyme to release RNA (mRNA), leaving DNA intact. Among various RNase isoforms reported, RNase H1 is highly specific. RNAi uses a small nucleotide sequence that includes RNase Argonaute 2 enzyme (AGO 2), which forms the RNA-induced silencing complex (RISC) that degrades the mRNA strand. The only difference between RNase and RNAi is that RNAi is associated with protein cleavage before interacting with the target site (Monia et al., 1993; Vickers and Crooke, 2015).

### 3.2 RNA interference

Over the last two decades, the role of RNA in the regulation of gene expression has attracted much attention. RNA interference (RNAi) is a novel and rapid gene silencing method in many organisms. The short RNA fragments silence the gene expression by inhibiting or degrading the mRNA that codes for the protein, thus preventing protein synthesis. The RNAi represents two types of small RNA molecules known as Small interfering RNA (siRNA) and Micro RNA (miRNA) that play an essential role in gene regulation (Agrawal et al., 2003).

#### 3.2.1 Small interfering RNA

**FIGURE 6:**
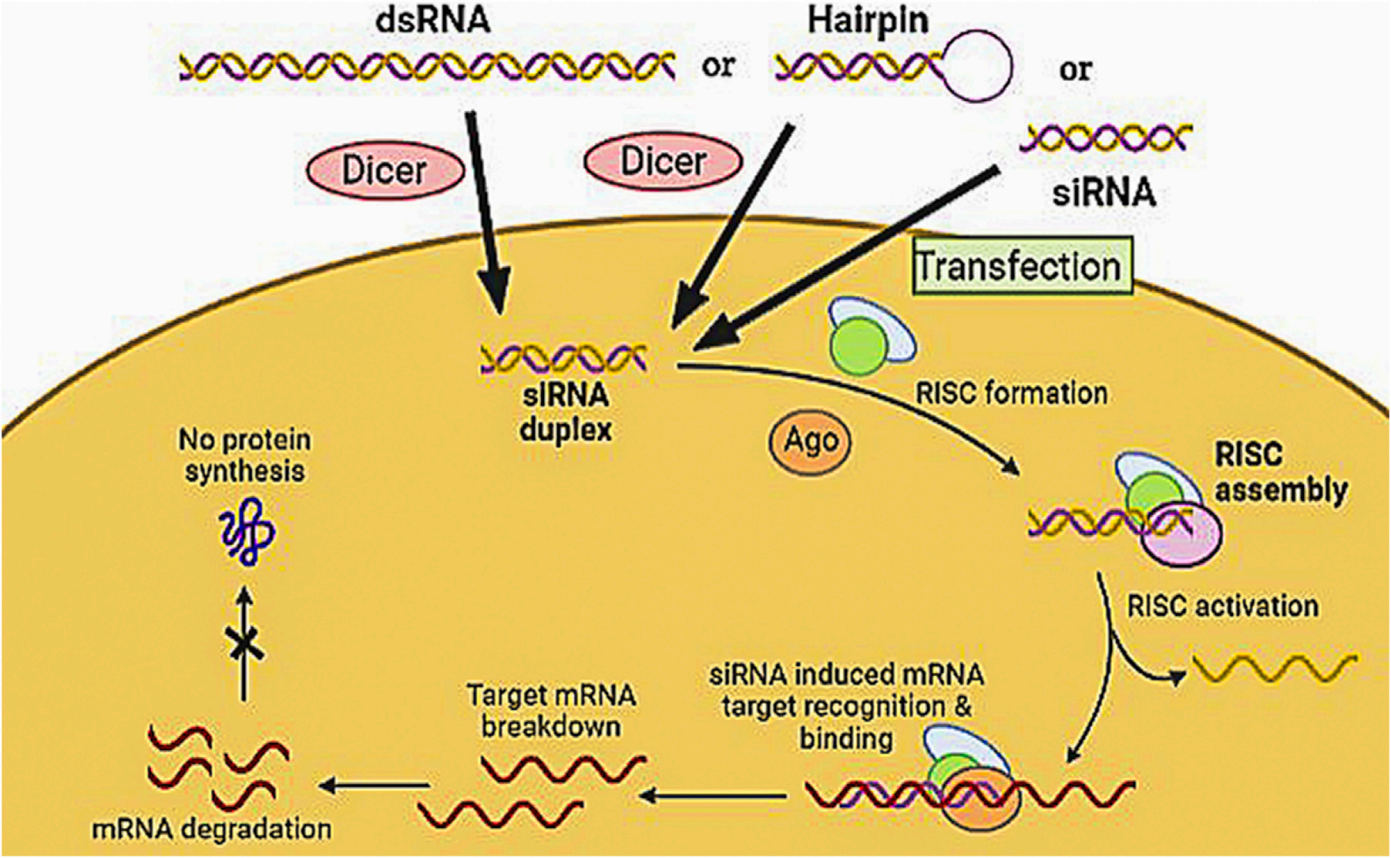
Mechanism of siRNA by assembly and activation of RNA- RISC. Formation of RISC followed by siRNA-RISC complex induced recognition and binding to mRNA target resulting in mRNA cleavage and sequence-specific gene silencing.

siRNA are short non-coding RNAs that uniquely regulate gene expression by acting specifically on mRNA targets. The mechanism of action of siRNA interference, as shown in Figure 6, is based on silencing genes at the post-transcriptional level and suppressing gene expression by mRNA degradation. siRNA consists of 19–21 nucleotides, with the TT and UU nucleotides largely overhanging at the 3′ end. The efficacy of double-stranded RNA (dsRNA) increases by lengthening it. The Dicer enzyme converts endogenous long dsRNA into siRNA, which blocks the expression of a target gene (Lam et al., 2015a). The two single strands of the cleaved dsRNA interact with endonuclease Argonaute 2 (AGO2) to form a small interfering RNA-induced silencing complex (siRISC). After the sense strand is released, the antisense strand is retained with the RISC. Then the activated RISC complex attaches to its target mRNA by a sequence complementary pairing which causes the degradation of the target mRNA by cleavage between the 10th and 11th nucleotide of a 50-end guide strand. These have a large molecular mass with a negative charge. Most of their aromatic nucleobase is inside the duplex, leaving just highly hydrated phosphates on the exterior (Juliano et al., 2008). Thus siRNA have the potential to be used therapeutically because they provide an opportunity for target-based selectivity. Currently, there are three siRNA approved by USFDA patisiran (2018), govisiran (2019), and lumasiran (2020) for rare metabolic disorders. Seven siRNA-based candidates are under Phase 3 clinical trials (Table 3.). Still, certain obstacles in siRNA drug development, i.e., low bioavailability due to their large size and anionic charge. Unfortunately, at the systemic level, delivery is compromised by rapid clearance through the kidney, and unmodified candidates exhibit a half-life of less than 5 min. The encapsulation of nanocarrier drugs is often subjected to serum protein absorption. Also, nuclease present in the tissue, plasma, and cytoplasm, degrades the siRNA. Thus, to tackle the site-specific delivery, two approaches enhance the clinical utility of siRNA drugs: chemically modifying the siRNA itself and designing various delivery strategies (Lam et al., 2015).

**TABLE 3:** Patient customized ASOs approved by US FDA.

S. No.	Strategy	ASO	Diseases
1	Modulation in splicing variants	Milasen	Batten Disease
2	Modulation in cryptic splicing variants	Atipeksen	Ataxia-telangiectasia mutation
3	Correction of splice	Jacifusen	ALS with FUS mutation

#### 3.2.2 Micro RNA

miRNA are 21–25 nucleotides long single-stranded RNA fragments that play a crucial role in regulating gene expression. These are small non-coding RNA molecules synthesized from parent miRNA using two RNase-II/III type proteins. They hamper the translation *via* accumulating mRNA (Dua et al., 2017). They degrade mRNA by acting on several targets and promote translational suppression, deadenylation, and decapping by attaching to a particular region in the 3′ UTR of its target mRNA. These binding sites are identified by the 5′ UTR mRNA region resulting in the silencing of the coding regions and gene expression. This miRNA-induced silencing complex (miRISC) contains the guide strand and AGO. The miRISC does complementary pairing on target mRNA called miRNA response elements (MREs). Thus miRNA degrades multiple mRNAs by acting on various targets, making them attractive drug targets with multifactorial diseases such as cancer and neurodegenerative diseases. Growing evidence suggests that it may contribute as a clinical biomarker for various diseases. However, miRNA drugs lag because they have not yet translated into FDA-approved candidates as siRNA, and none have entered the phase 3 trials. There are only two miRNA therapeutics in the early phase of the study for cancer treatment. MRX34 is the first candidate to enter a clinical trial for miRNA replacement therapy using liposomal formulations to mimic miRNA-34. It is a naturally occurring tumor suppressor gene that inhibits the signaling cascades in liver cancer. Another candidate, TargomiRS, mimics the miRNA-16, which inhibits tumor-promoting gene transcription. The primary drawbacks of miRNA are delivery to the CNS and its stability, similar to siRNA; both have inherent negative charge properties, hydrophilic nature, and high molecular weight (Wahid et al., 2010). Thus, both RNAi therapeutics—siRNA and miRNA—hold a great place as a new therapeutic class for gene silencing in the near future.

### 3.2 CpG oligonucleotides

These are single-stranded, small oligonucleotides with unmethylated CpG dinucleotides in a specific area. CpG oligonucleotides act as an immunostimulant to cells that express Toll-like Receptor nine (TLR 9), such as macrophages, B cells, dendritic cells, and monocytes. These are classified into four groups based on structural differences and the immunological response they elicit (Carpentier et al., 2003).

1) K-type/B-type structure consists of a phosphorothioate backbone having multiple CpG motifs. They stimulate plasmacytoid dendritic cell (pDC) maturation to trigger B cells for the production of IgM and TNF differentiation,
2) D-type/A-type structure comprises a mixed backbone encompassing a phosphodiester nucleotide core and phosphorothioate terminals with a single CpG motif. They stimulate pDC maturation and IFN-secretion, as well as APC maturation,
3) C-type structure consists of a phosphorothioate backbone with multiple CpG motifs embedded in a central palindrome that forms either dimers or a stem-loop-like structure. It induces the proliferation and differentiation of B-cell and pDC, as well as IL-6 and IFN-production, and
4) P-type CpG structure consists of a phosphorothioate backbone with numerous CpG motifs and double palindromes that make a hairpin-like structure rich in 3′ends GC and stimulate pDC, B-cells, and IFN-α secretion (Shirota et al., 2015).

### 3.3 Aptamers

**FIGURE 7:**
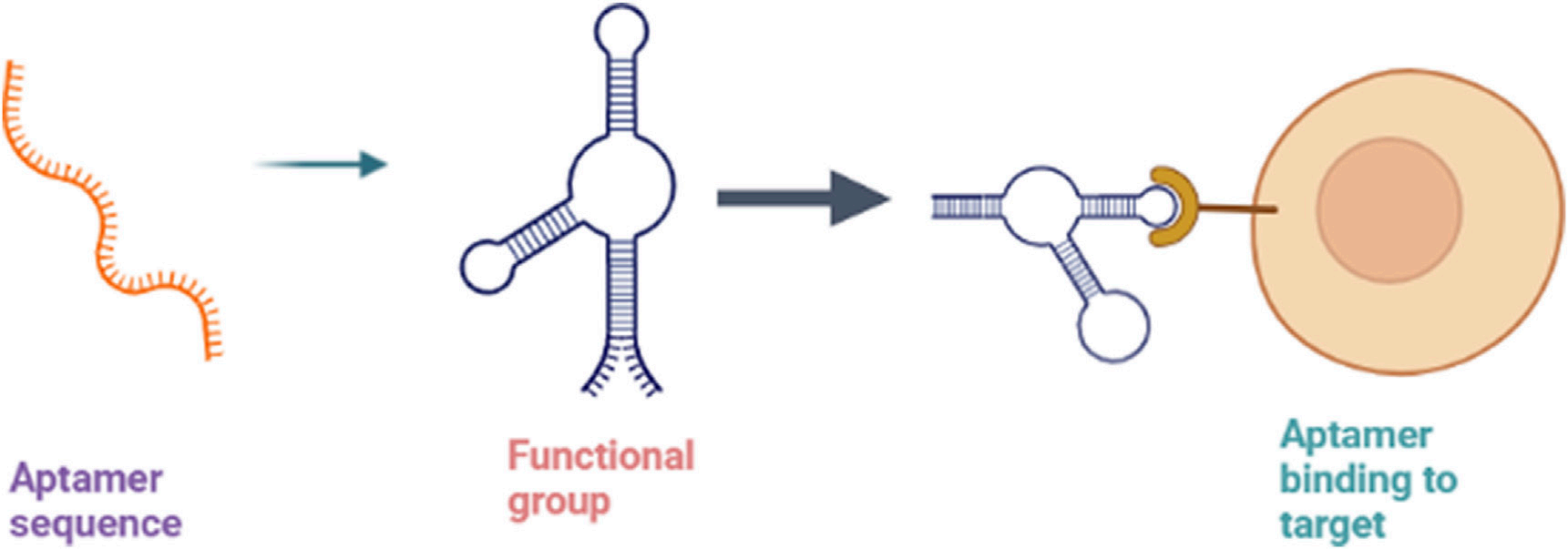
Mechanism of aptamers showing sequence folding into 3D structure formation (functional aptamer) and binding to the target.

The term “aptamers” refers to the class of short synthetic RNA or DNA oligonucleotides or peptides that selectively bind to their target site with high specificity and affinity, often inhibiting protein-protein interactions. The activity of aptamers is based on their potential to build three-dimensional structures with a target rather than binding with nucleic acids (Figure 7). While usually, the size of the aptamer sequence varies from 15 to 60 nucleotides, the most functional size is in the range of 40 nucleotides. Unmodified aptamers have half-lives of 2 min due to degradation by nucleases (Song et al., 2008). They act on various targets, including proteins, peptides, metal ions, metabolites, toxins, carbohydrates, bacteria, viruses, living mammalian cells, and small organic molecules. The aptamer interaction with the target is usually mediated by Van der Waals forces, electrostatic interactions, hydrogen bonding, stacking interactions, and shape complementarity (Li and Lan, 2015). The aptamer flexibility allows them to be wrapped around the small target molecule and fit into the clefts and spaces within the surface of the large target molecules. The three-dimensional structural features of aptamers, such as hairpins, pseudoknots, G tetramers, bulges, and internal loops, aid in the binding of an aptamer to the target site (Klussmann, 2006).

Aptamers may be nucleic acid analogs of specific antibodies, as their interaction with the target is similar to antigen-antibody interactions (Söylemez and Türkseven, 2019). Their target recognition, synthesis, and optimization surpass antibodies in target specificity, affinity, ease, and cost-effectiveness. Although aptamers are equivalent to specific antibodies, they possess superior features, like good chemical and thermal stability, longer shelf life, and large-scale high-speed production while maintaining high reproducibility and reliability. Unlike monoclonal antibodies, they can be made using animal-free procedures and attach to toxic and non-immunogenic antigens (Michaud et al., 2003). They are comparatively smaller (8–15 kDa), resulting in better tissue penetration and cellular internalization required explicitly in solid tumor therapy. Compared to small-molecule ligands, aptamers have several advantages like tailored specificity and affinity towards an unlimited number of targets, minimal toxicity, high stability, low batch-to-batch variation, and production scalability. The various advantages of aptamers have led to their widespread use in biomedical fields such as diagnostics, imaging, biosensing, biomarker detection, and targeted therapy (Haßel and Mayer, 2019).

Currently, the application of aptamers is investigated in cancer, obesity, diabetes, cardiovascular diseases, age-related disorders, autoimmune diseases, bone diseases, and bacterial and viral infection therapies. The use of aptamers in the detection and treatment of viral infections continues to attract attention due to their high efficacy, minimal/no side effects, low risk of drug resistance, and immediate social impact (Kaur et al., 2018). Ongoing efforts focus on combining different aptamer modules with other groups such as cholesterol, nanoparticles, reporter groups, chemotherapeutic molecules, siRNA, and ASOs to develop multivalent aptamers with improved characteristics and stability. Among the available chemical conjugate approaches, nucleic acid aptamers are of prime interest as they provide a considerably more suitable substrate for developing multivalent aptamers. For instance, RNA aptamers for targets like HER3/ERBB3, OX40, 4-1BB, CD40, and CD28 have been developed, while DNA aptamers for targets like human VEGFR-2 and insulin receptors have been examined (Keefe et al., 2010). In 2004, pegaptanib, an anti-angiogenic NX1838 aptamer specific for a vascular endothelial growth factor (VEGF), was approved by the US FDA for treating ocular vascular disease (Siddiqui and Keating, 2005).

### 3.4 Clustered regularly interspaced palindromic repeats

CRISPR is a group of DNA sequences found in prokaryotes like bacteria and archaea that have been known to constitute the prokaryotic adaptive immune system against the infecting bacteriophages (Barrangou and Doudna, 2016). CRISPR-associated protein 9 (CAS 9) is a restriction endonuclease that uses CRISPR strands as hybrids to identify and cleave DNA strands complementary to CRISPR sequences, mediating genome modification (Barrangou, 2015). CRISPR-CAS 9 gene editing technology permits genome correction and knock-down or knock-in of genes with relative efficiency and specificity (Sahel et al., 2019). The therapeutic applications of this restriction system are being studied in various diseases like Alzheimer’s, HIV, sickle cell anemia, cancers, Leber Congenital Amaurosis, and other neurological disorders. Currently, CRISPR-CAS-based treatments are in the early stages of clinical trials for conditions including cancers, chronic infections, eye-related diseases, and blood and protein folding disorders (Abudayyeh and Gootenberg, 2021).

### 3.5 Decoys

Decoys are transcription factor targeting, single-stranded RNA-based oligonucleotides that consist of a 2′-O-methyl alteration in the ribose sugar moieties of the nucleotide strand (Crinelli et al., 2002). It confers stability to the oligonucleotide molecule, making them a powerful strategy in gene therapy (Mann and Dzau, 2000). When delivered intracellularly, decoys with the specific consensus binding sequence belonging to a particular transcription factor are recognized by the target factor as it binds to them. Also, it impedes the protein’s ability to attach to the promoter regions of the disease-causing target gene and subsequent deletion of the trans factor that ultimately results in the reduction or complete blockade of gene expression (Denichenko et al., 2019). Similarly, therapeutic decoys can be designed to stimulate gene expression that has been suppressed transcriptionally. Presently, decoy-based oligonucleotides for use in auto-immune and inflammatory diseases are in Phase III clinical trial stages in Japan and United States (Igarashi et al., 2022).

## 4 Advantages of oligonucleotide therapy

OT have seen significant development substantially due to their potential to treat or manage a wide range of diseases. Because of target specificity, it has become meaningful therapeutics for clinical productivity. The structure and basic sequence of OT directly impact their delivery and potency (Mollocana-Lara et al., 2021).

Various advantages that led to rapid growth and recognition of ON drugs are:

1) Oligonucleotide medicines can modulate a variety of disease targets, including more than 10,000 proteins in the human genome that have previously been thought to be undruggable by small molecules or protein therapies (Yin and Rogge, 2019);
2) Identification of drug candidates requires only the identification of a target sequence on the RNA that is part of the disease pathogenesis (Gredell et al., 2008);
3) The inherent ease and speed of synthesizing new libraries of complementary oligonucleotide sequences. Active pharmacophores can be identified in weeks, and the corresponding drug is produced speedily (Wadhwa et al., 2020);
4) Oligonucleotide drugs are widely used to treat neurodegenerative diseases (like amyotrophic lateral sclerosis, Huntington’s disease, Spinal muscular atrophy), respiratory disorders (like asthma and COPD), diabetic retinopathy, and many cancers. Specific structural modifications such as changes in nucleotide backbone or sugar moieties of the oligonucleotide therapeutic may introduce desirable features into the drug molecule which can improve its safety and efficacy (Zhang et al., 2021);
5) When compared to small molecules, the relative cost-effectiveness of designing, formulating, manufacturing, and purifying OT at large industrial scale is more efficient (Burnett and Rossi, 2012);
6) A plethora of simple and reliable manufacturing platforms are available, ranging from batch plasmid DNA bioprocessing and cell-free enzymatic synthesis to entirely chemical synthesis (Kher et al., 2011);
7) Enhanced safety profile and fewer regulatory requirements of many oligonucleotide drugs, including RNA therapeutics like mRNA-based vaccines and cell therapies (Fiset and Gounni, 2001).

## 5 Limitations and challenges to oligonucleotide therapy

Despite their great therapeutic potential, there are several limitations encountered with OT (Fiset and Gounni, 2001). The major problems faced are toxicity, drug delivery, and uptake. For instance, phosphorothioate-modified oligonucleotide (PS-ODNs) treatment reduced platelet counts leading to thrombocytopenia (Frazier, 2015). Toxicological problems may be caused through the sequence and chemistry or are most likely due to the binding of ON to the proteins on the cell surface or in the serum. PS-ODNs tend to activate the complement cascade events and lead to changes in heart rate, blood pressure, and cardiac output (Vaerman et al., 1997). In various preclinical studies, immunostimulatory effects have also been associated with many of the siRNAs and antisense oligonucleotides. Another problem is degradation by nucleases, particularly of natural oligonucleotides composed of ribose or deoxyribose sugars with phosphate backbone and bases. Further, nucleotide-5′ monophosphates, a degradation product of the same, can inhibit cell growth and proliferation (Magen and Hornstein, 2014).

Similarly, oligonucleotides such as double-stranded RNAs and naked siRNAs are degraded or fail to enter the cells and are rapidly cleared from circulation even before they show their activity. The limited uptake and transport through cell membranes of oligonucleotides are attributed to their polarity and intrinsic physicochemical properties. Specific and efficient delivery of the oligonucleotide to the target tissues, primarily through the blood-brain barrier (in neurodegenerative diseases), is also challenging to achieve (Khvorova and Watts, 2017). Other major obstacles in using OT are off-target interactions and saturation of endogenous RNA processing pathways (Wan et al., 2015). Numerous obstacles still exist before the use of oligonucleotide therapies in clinical settings.

To develop ON drugs, potency, specificity, and efficacious delivery issues must be addressed (Robbins et al., 2009). Specifically, the following safety aspects must be addressed.

### 5.1 Persistent gene silencing

RNA-induced silencing complex is used in the siRNA strategy, which results in persistent post-transcriptional gene knockdown for up to 2–4 weeks. This long-term gene silencing allows siRNA therapies to be administered less often (Voutila et al., 2017). However, without the injection of an antidote, multiple mechanism-based harmful and off-target side effects can last for weeks (Klabenkova et al., 2021).

### 5.2 Off-target related toxicities

The off-target toxicities may arise with oligonucleotide therapeutics if the oligonucleotide sequence is strictly complementary to the target sequence, partial similarity with genes on other unintended target sequences may induce off-target effects that may lead to downstream changes in gene expression (Jason et al., 2004).

### 5.3 Immuno-stimulatory responses

Several oligonucleotides are reported to attach to toll-like receptors (TLRs) that play a vital role in the innate immune system and subsequently induce immune responses identical to those stimulated by viral and bacterial nucleic acids (Judge and Maclachlan, 2008). Oligonucleotides such as ssDNA that are RNase H-dependent or splice altering or those with specific sequence motifs are found to be significant agonists of TLRs. For example, using single-stranded phosphorothioate oligonucleotides has often been associated with injection site reactions, fever, chills, and rigors at high doses (Rayburn and Zhang, 2008). Similarly, lipid-nanoparticle formulations of siRNA are found to promote inflammatory responses as they induce complex antiviral-like innate immune responses. Adaptive immune responses have been more muted comparatively (Iannitti et al., 2014).

### 5.4 Renal accumulation, thrombocytopenia, and inhibition of coagulation

Mild to low proteinuria and rare cases of glomerular nephritis have been reported in some patients on phosphorothioate oligonucleotide therapy. Fluctuations in protein, creatinine, and plasma urea levels after treatment with phosphorothioate oligonucleotides are also reported (Robbins et al., 2009). Phase I clinical trial of the phosphorothioate at 5 mg/Kg of body weight per week indicated acute tubular necrosis. This may be due to the renal accumulation of the single-stranded, phosphorothioate oligonucleotides that are usually protein-bound and avoid glomerular filtration (Huang, 2017). The rest of the unbound fraction is rapidly reabsorbed by renal proximal tubular cells. Although renal toxicity is observed to be sequence-specific, it has led to stricter regulatory surveillance. Some oligonucleotides have also been associated with thrombocytopenia in small groups of patients in clinical trials (Henry et al., 1997). 3% of patients receiving long-term treatment of Drisapersen (6 mg/Kg per week) and patients on phosphorothioate oligonucleotide regime for triglyceridemia and transthyretin amyloidosis reported having experienced a rapid drop in platelet count leading to class 4 thrombocytopenia. Certain ASOs and siRNAs have been found to inhibit blood coagulation by activating the complement cascade and blockade of the intrinsic pathway for clotting (Crooke, 2017).

### 5.5 Oligonucleotide instability and delivery-related issues

Apart from the instability of oligonucleotides against enzymatic degradation, safe and efficacious delivery remains the greatest challenge to the clinical development of oligonucleotide therapeutics. Since oligonucleotides are heavy and negatively charged macromolecules, when administered in their unmodified/naked form, they come across many barriers that hamper their absorption, disposition, and therapeutic activity (Sepp-Lorenzino and Ruddy, 2008). Even in the case of RNA therapeutics given intravenously, the circulating RNases swiftly degrade the naked and unmodified RNAs. Systemic delivery of the macromolecule to most organs and tissues except the liver has proved to be a significant roadblock to their use (Mehta et al., 2019).

## 6 Strategies to overcome the challenges faced with oligonucleotide therapy

Uptake and delivery of the oligonucleotide therapies remain the most significant challenges. After parenteral administration, oligonucleotide drugs must travel through the bloodstream, pass through the biological membranes, and be taken up by the cells. They must avoid lysosomal and enzymatic degradation and bypass entrapment in secretory vesicles. To overcome these problems, various strategies are employed to enhance the stability of oligonucleotide therapies and promote their delivery to target tissues (Lysik and Wu-Pong, 2003). The following three strategies are widely used.

### 6.1 Chemical modification strategies

Bare and naked oligonucleotides are most prone to degradation and have poor-drug-like properties. Chemical alteration is one of the most effective ways to improve the delivery of oligonucleotide drugs. Modifying the sugar motif or changes in the phosphate backbone of the oligonucleotide can keep them metabolically stable, and functional, enhance their protein-binding properties and delay their renal clearance. Each alteration adds diverse features to the oligonucleotide, some of which might be a combination, complicated synthesis, or hinder its mechanism of action (Chen et al., 2005). The type of chemical modification to be introduced into the oligonucleotide depends on the target.

First-generation oligonucleotides had alteration of the phosphate backbone, for example, phosphorothioate modified oligonucleotides where Sulphur atom replaces an oxygen atom in the phosphate moiety to impart resistance to the molecule against endonuclease and improve its bioavailability by decreasing the renal clearance (Corey, 2007). This change, however, not only diminishes the affinity for the target, but also imparts toxicities.

The 2′-OH position of the RNA and the 2′ position of the DNA are modified in second-generation oligonucleotides. e.g., 2ʹ-O-methyl and 2ʹ-fluoro modifications improve the binding affinity of the oligonucleotide to the target RNA and further increase its nuclease resistance (Kolate et al., 2014). Conformationally restricted DNA analogs like tricyclo-DNA (tcDNA) and locked nucleic acid (LNA) tend to have an even greater binding affinity. Between the 2′-O and 4′ carbon positions of the ribose ring, LNA has a methyl bridge, and between the 3′ and 5′ carbon positions of the ribose sugar, tcDNA consists of an ethylene bridge with a cyclopropane ring. Their locked conformation makes them ideal for binding to RNA (Geary et al., 2015).

**FIGURE 8:**
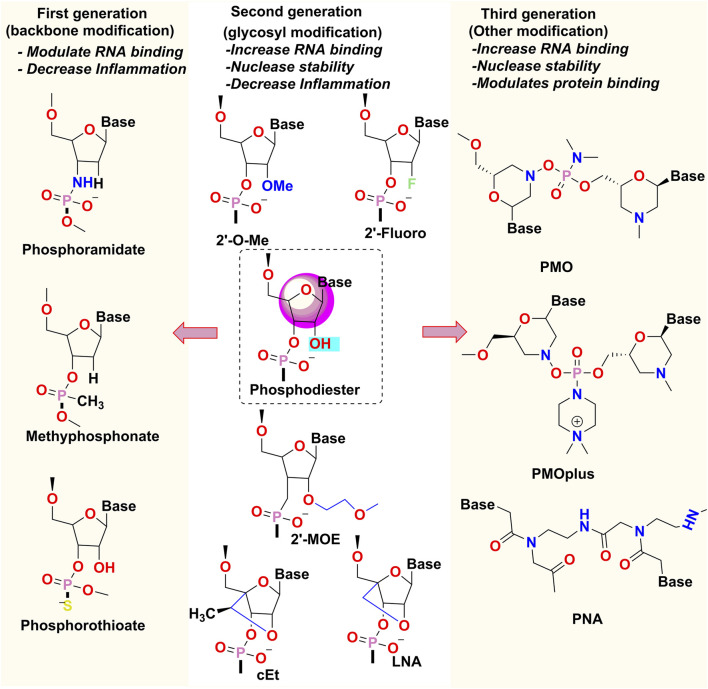
Chemical modifications of Oligonucleotides- 2′-OMe: 2′-O-methyl, 2′-MOE: 2′-O-methoxyethyl, LNA, locked nucleic acid; cEt, constrained ethyl bridged nucleic acid; PMO, phosphorodiamidate morpholino-oligonucleotide; PNA, peptide nucleic acid.

Third-generation oligonucleotides involve modifications of the nitrogenous bases that form the oligonucleotide polymer. For example, Phosphorodiamidate morpholino-oligomers (PMO) have morpholine ring with phosphorodiamidate attachments in place of the nucleic acid backbone and peptide nucleic acid (PNA), whose nucleobases are linked by amide bonds instead of phosphodiester bonds (Figure 8). Both these oligomers are charge less, resistant to degradation by nucleases, and exhibit variable affinity for the target RNA sequence. (Mollocana-Lara et al., 2021).

### 6.2 Conjugation and targeting

Conjugation of therapeutic oligonucleotide molecules with different chemical moieties can improve the ADMET properties of the oligonucleotide and direct it to the target tissues. Chemical conjugation can be achieved by linking the oligonucleotide with entities such as polymers, peptides, lipids, antibodies, receptor ligands, and aptamers.

Polyethylene glycol (PEG) is a very useful polymer, categorized as “Generally Regarded as Safe (GRAS)” by the FDA. PEG is a very flexible, uncharged, hydrophilic polymer that protects the conjugated drug from nuclease degradation by forming a hydration shell, thus preventing the interaction of the drug entity with another biomacromolecule (Kolate et al., 2014). PEGylation also increases the circulation half-lives by decreasing renal elimination and improving oligonucleotide stability.

Antibody-RNA conjugates such as monoclonal antibodies and functional oligonucleotides consisting of antibody fragments are increasingly being used for biomedical imaging and protein identification. Antibody conjugates are also used as a carrier for many oligonucleotide therapies (Nanna et al., 2020). For example, the intracellular transport of the iron-transferrin complex is mediated by an antibody fragment particular to the transferrin glycoprotein receptor, which has been used to direct siRNA towards skeletal muscles and cardiac tissues. Similarly, ASOs in conjugation with anti-CD44 monoclonal antibody may be used to reduce the expression of DRR/FAM107A, a major protein involved in glioblastoma cancer (Sugo et al., 2016).

Lipid conjugating therapeutic oligonucleotides with hydrophobic molecules like cholesterol can enhance endosomal release, resulting in a longer plasma half-life. Such conjugations improve drug delivery to the liver and peripheral tissues such as muscles by passive targeting (*via* enhancing the binding affinity to blood plasma proteins) and active targeting (*via* endogenous lipid transport) (Osborn and Khvorova, 2018).

Peptide conjugates are usually short cationic or amphipathic peptides (less than 30 amino acids in length) that can translocate oligonucleotide drugs across various biological barriers and membranes by direct penetration or by interacting with specific receptors. Once internalized, these cell-penetrating peptides become fully functional and active, resulting in endosome disruption and drug release into the cytoplasm. For example, Pip6a, a cell-penetrating peptide (CPP) is made up of a hydrophobic core bound by arginine-rich entities. In conjugated form, it exhibits serum stability and easily crosses plasma and endosomal membranes (Lindgren et al.;Bechara and Sagan, 2013). However, the intriguing benefit of CPPs is that they are direct conjugates with a positive charge and neutral oligonucleotides like PMO and PNA. The peptide PMO conjugates are utilized to treat a variety of disordered, e.g., DMD (dystrophin exon skipping) (Yin et al., 2008).

Tissue-specific targeting may be attained by conjugating the oligonucleotides with receptor ligands that allow attachment of the drug to the receptors located on the target cell locations and mediate tissue-specific distribution. The different types of receptors that can undergo conjugation include carbohydrates, peptides, antibodies, aptamers, and other receptor-ligand systems (Richard et al., 2003). One of the most popularly known targeting ligands is Acetyl galactosamine (GalNAc) which binds to the asialoglycoprotein receptor 1 (ASGR1) highly expressed in hepatocytes. GalNAc in conjugation with oligonucleotides like siRNA facilitates the drug’s highly specific and efficacious delivery into the liver cells. Preclinical studies in a mouse model have shown that a GalNAc-antisense oligonucleotide targeting human transthyretin was ten times more potent and long-acting than unconjugated antisense oligonucleotide (Ming and Laing, 2015).

Aptamer conjugated oligonucleotide drugs are easy and cost-effective to design, conformationally flexible, have low immunogenicity, confer nuclease resistance and improve their blood plasma half-life (Huang, 2017). The first aptamer-siRNA conjugates were intended to deliver apoptosis-inducing siRNAs to prostate-specific antigen-expressing cancer cells. In preclinical cancer models, aptamer has shown to be successful in *in vivo* distribution of miRNAs, antisense oligonucleotides, antagomirs, and bi-modular miRNA-antagomirs (Odeh et al., 2019).

### 6.3 Delivery vectors

The properties required in an oligonucleotide can be tailored by formulating targeted advanced drug carrier systems. The ADME characteristics of the carrier-based delivery compositions are mostly dependent on the features of the delivery systems and independent of the oligonucleotide. The type of targeting may be passive or active (Juliano et al., 2008; Asami et al., 2016). Particulate carrier-based delivery vectors improve intracellular passage and delivery by increasing cellular utilization and endosomal escape. Therefore, the concentration of drug at the non-target location is reduced contributing to lower toxic effects and the concentration reaching the desired target increases, resulting in a better therapeutic index. A plethora of delivery systems are available that are either natural-based or synthetic-based and display great potential in delivering the oligonucleotide to the target of interest (Lebedeva et al., 2000). Many polymeric nano-drug delivery-based vehicles have been investigated, like DNA nanostructures, gold nanoparticles, mesoporous silica, exosome-like nanocarriers, spherical nucleic acids, etc., but the poor plasma clearance of these nanoparticles from the liver makes direct delivery to other tissues and organs difficult (Akash et al., 2016).

The various delivery systems of interest that enhance the oligonucleotide drug delivery system are as following:

#### 6.3.1 Lipid-based delivery systems

The cationic lipids entrap the negatively-charged oligonucleotides with strong electrostatic interactions and serve as highly efficient carrier systems. However, it is often associated with dose-dependent systemic toxicity, due to which ionizable lipids that are positively charged at acidic pH are preferred [79]. The examples include lipidoids and D-Lin-MC3-DMA, having multiple amines that undergo protonation in an acidic environment and remain unchanged at pH 7. The hydrophobic lipid tails stabilize the overall oligonucleotide arrangement in the formulation by engaging in hydrophobic interactions. Exosomes are also lipid-based nano-vectors. These microscopic vesicles are shed from cells, encapsulating a little portion of the cytoplasm in the process. They are proven to be highly biocompatible and have the potential for highly selective active targeting of endogenous cellular ligands. But the main disadvantages lie in the production of large-scale reproducible batches with effective drug-loaded exosomes and heterogeneity in their composition (made up of natural contents of proteins and nucleic acids derived from the target cell) (Ma et al., 2002; Wang et al., 2015).

#### 6.3.2 Peptide-based delivery systems

The cell-penetrating peptides (CPP) can also be used as a carrier-based drug delivery system. The stability of CPP-oligonucleotide nanocomplex is governed by the electrostatic and hydrophobic type of interactions between the cationic peptides and anionic oligomers. In comparison to conjugated CPP-oligonucleotides, peptide-based delivery systems are considerably more amphipathic and incorporate additional chemical alterations to the CPP sequence like hydrophobic fatty acid segments to the CPP sequences that enhance the efficiency of delivery to the target site and stability of the oligonucleotide in the formulation (Mäe and Langel, 2006).

#### 6.3.3 Antibody-based delivery systems

Antibodies, apart from being used in their conjugated forms, are also used as carrier delivery systems where the antibodies and antibody fragments are fused with either avidin or protamine groups. Here, antibody–avidin molecules are fused with biotinylated oligonucleotides to achieve target-specific delivery. The cytotoxic siRNAs were conjugated with Her2-positive cancer cell-specific antibodies using peptide protamine linked to siRNA (Pardridge and Boado, 1991; Dugal-Tessier et al., 2021).

#### 6.3.4 Polymer-based delivery systems

Polymer-based systems are potential carriers of oligonucleotide therapies, as they confer chemical flexibility, high structural stability and stability during storage, and monomeric sequences and side group functionalities can be engineered. Polylactic-co-glycolic acid (PLGA) is one such highly biocompatible copolymer that is being explored. Encapsulation of small anionic oligonucleotide drugs in cationic polymeric nanoparticles can be achieved (Xiao et al., 2020). For this purpose, cationic polymers like poly (amidoamine), poly (propylene imine) and poly (L-lysine) may be used. A hyperbranched class of polymers, namely dendrimers, may be used to complex with many oligonucleotide molecules. Apart from these, synthetic polymers like polyphosphazenes are known to be highly biocompatible and chemically flexible promising vectors used to deliver therapeutic oligonucleotides. Recently, lipid–polymer hybrid nanoparticles (LPHNs) having dual properties of lipid and polymer nanoparticles, are reported to confer serum stability and biocompatibility to the oligonucleotides (Hammond et al., 2021). In general, LPHNs consist of a polymer-based coat surrounding the oligonucleotide therapeutic, which is, in turn, encapsulated by a lipid-based exterior. Biodegradable polymers like polylactic acid, chitosan, polycaprolactone, etc., may be used to form the polymeric core, and the lipidic shell’s composition could be modified based on the therapeutic agent to confer desirable drug release, cellular uptake, and biodistribution. Another carrier-based approach that overcomes the limitations of lipid and polymer delivery systems is called lipopolyplexes. These carriers have dual properties of polyplexes and lipids, exhibiting superior systemic stability, efficient drug delivery, reduced cytotoxicity, etc. Lipopolyplexes are nano complexes composed of lipoplexes, polycations (peptides or polymers), and nucleic acids. Due to their high specificity, biocompatibility and versatility, lipopolyplexes are being developed for different therapeutic needs (Rezaee et al., 2016; Jerzykiewicz and Czogalla, 2021).

## 7 Pharmacokinetics of oligonucleotides

The success of new therapeutics depends upon their pharmacokinetic properties (PK) such as adsorption, distribution, metabolism, elimination, and safety profile (Courtenay, 1998). Oligonucleotides are majorly administered *via* parenteral routes—either intravenous infusion or subcutaneous injection. After subcutaneous administration, these therapeutics are rapidly absorbed and distributed in blood circulation with plasma peak concentration within 3–4 h. The possible mechanism of cellular uptake is facilitated by endocytosis. The ONs with phosphorothioate backbone are found to be abundantly bound to plasm proteins (more than 90%), whereas low-affinity with albumin reduces plasma half-life to 1–2 h (Geary, 2009; Herkt and Thum, 2021). ONs such as PNAs, and morpholinos lack charge due to which they are poorly bound to plasma proteins; hence, get rapidly cleared by metabolism in blood or urinary excretion. Due to the polar backbone these therapeutics do not cross the blood-brain barrier (BBB), but they can be locally given into the cerebrospinal fluid (CSF) site and the spinal cord with longer residence time and reduced nuclease activity (Geary et al., 2015). Similarly, siRNA which are considered to be polyanionic in their unmodified or unformulated form are very unlikely to interact with the negative charge of the cytoplasmic membrane. Moreover, the delivery of siRNA *via* endosome will not ensure it release and will eventually undergo the hydrolysis influenced the low pH. Due to this naked siRNA has a very short half-life (t_1/2_) of only 2–6 min which is further influenced by hydrolysis mediated by RNases within seconds upon injection. In alignment with ONs, they also possess poor plasma protein binding and are concentrated chiefly in kidneys, thus lacking in attaining a minimum effective concentration and, consequently, a pharmacological response (Gallas et al., 2013).

## 8 Patient’s customization

In recent years, personalized customization has emerged as an alternative to address the lack of specificity and provide optimal safety, efficacy, and thereby improved quality of life for an individual (Oberthuer et al., 2006). Due to phenotypic and genotypic differences, a drug that is safe for one patient can be ineffective or even harmful to another. It has been observed that patients suffering from rare diseases (RD) are a small population with severe life-threatening complications compared to other common diseases. These diseases have fewer treatment options available. Because 80% of RD are genetic, rare cancers, some tropical infections, and degenerative disorders are significant challenges in these cases. As a result, there is no universal definition for an RD; different countries define it differently based on their population, resources, and health system. RD affects approximately 6%–8% of Europeans; in the United States, about 30 million are affected by rare diseases. Furthermore, a more significant impact is seen in children; about 50% of cases and 35% of deaths upto1-year old, 10% are between 1 and 5 years, and 12% are in the age group of 5–15 years (Lochmüller et al., 2018; Fyfe, 2019). There is an urgent need for therapeutic options for these patients, and personalized treatment has been shown to improve the quality of life. Because of the urgency of the patient’s clinical situation and the possibility of sequence-specific of customization, ASOs are now the third major class of drugs in the discovery pipeline, after small molecules and biologics. ASOs identify the genetic mutation in a wide range of rare, fatal neurodegenerative diseases in deep intronic, which leads to aberrant pre-mRNA splicing. But later research indicates that mutation-induced incomplete splicing can be fixed (Schoch et al., 2016; Lim et al., 2020). In this section, the successes of some ASOs as the customized therapeutic option for rare diseases are discussed:

1) Batten’s disease:

Milasen is the first patient-customized ASO, specifically developed to treat a 6-year-old girl named Mila with Batten’s disease, also known as Neuronal Ceroid Lipofuscinosis 7 (CLN7). It is a fatal neurodegenerative condition that affects CNS and retina, leading to blindness, dysarthria, ataxia, frequent falls, and dysphagia. The girl’s whole-genome sequencing (WGS) revealed a mutation in the MFSD8 intron 6, resulting in incorrect splicing. For targeting intron 6, seven ASOs were designed, which activated the cryptic 3′ splice site among all three, leading to increased splicing. After chemical modification of nusinersen, it was found to be safe. This ASO, called Milasen as per the girl’s name, is a 22-nucleoside oligonucleotide having phosphate linkages and phosphorothioate and 2′-O-methoxyethyl (2′-OME) sugar moiety. It more than tripled the amount of normal splicing, lessening the frequency of daily seizures and temporarily improving the patient’s overall quality of life. Thus, Milasen is a genomic medicine used for personalized treatment for individuals when no therapies are available at the time of diagnosis (Kim et al., 2019).

2) Amyotrophic Lateral Sclerosis (ALS):

Another study was conducted on patient Jaci Hermstad, a 26-year-old suffering from ALS caused by a mutation in the Fused in Sarcoma (FUS) gene. ALS is a rare, fatal neurodegenerative disorder characterized by the loss and dysfunction of neurons in motor pathways. This was the first case to be treated with newly designed antisense therapeutic ION363 that decreased the FUS protein production and prevented disease progression. ION363 is also named Jacifusen in honor of the patient. The BIIB067 is currently a drug that is under Phase 3 trial for ALS with superoxide dismutase 1 mutation (SOD1) (Kim et al., 2020).

3) Ataxia-telangiectasia:

ASO have been developed for various other patients *via* regulatory pathways such as for ataxia-telangiectasia (AT) mutation, a rare inherited disorder that affects the central nervous system, immune system, and other parts of the body. The AT mutation appears in infancy or early childhood to treat this complex neurodegenerative disordered experimental drug showing the promising result is Atipeksen. Atipekesen targets the correction of the cryptic splice-acceptor site under investigational new drug (IND) by the FDA (AA et al., 1999; Synofzik et al., 2022).

### 8.1 Targeting of pathogenetic variants by splice modulating antisense oligonucleotides

Next-generation sequencing provides patients with an accurate molecular diagnosis as it identifies the genetic causes of disease through genome sequencing. Such genetic variations have been targeted to provide therapeutics, even if these benefit a single patient (Muir et al., 2016; Owen et al., 2021). This is an important reason for using ASOs as a tool for customized individual therapy. ASOs can modulate splicing by inducing exon inclusion, exon skipping, modulating alternative splices, or correcting cryptic splice variants to restore protein production. The design and optimization of ASOs capable of splice modulation is still a matter of hit and trial (Lemmers et al., 2012).

The splice modulating ASOs target various pathogenic variants as follows:

#### 8.1.1 3 Deep intronic splice variants

These variants have the potential to influence alternative splicing by interfering with splice recognition and are excellent targets for ASO-mediated splice modulation. The intronic variants cause the skipping of one or more cryptic exons and restore the normal protein. These variants also affect the canonical donor or acceptor splice sites (Hoffman et al., 1988).

#### 8.1.2 Reading frame disturbance variants

These variants within exons can disrupt the reading frame. Thus, to generate an inframe transcript of a disrupted exon, the ASO-mediated multiple exon skipping can restore the reading frame and allows the production of an internally deleted partially functional protein (Bremer et al., 2019).

#### 8.1.3 Toxic gain-of-function variants

These pathogenic variants can lead to the generation of toxic proteins. Shorter nontoxic proteins will be formed by skipping of the exon harboring the variants (Aartsma-Rus et al., 2017).

#### 8.1.4 Missense variants

Missense variants of the crucial domains of proteins are categorized based on the likelihood of generation of GOF (gain-of-function) and LOF (loss-of-function) genes (Lim et al., 2020). Thus, GOF and LOF genes formed from the loss-of-molecular-function (LOMF) variant and gain-of-molecular-function (GOMF) can form the basis for a systematic approach to treatment. Following are the results of these variants, which may be contrasting at times (Bell et al., 2019):

##### 8.1.4.1 Loss-of-molecular-function variants resulting in the loss-of-function

These variants are caused by deletion of the gene or frameshift mutations leading to loss of function of the gene. Such variants are treated by gene replacement therapy. But, if there is one active functional copy of the gene, ASOs can play a role by three mechanisms to upregulate the expression level of a healthy gene: 1) by promoting poison exon (PE) skipping. PEs are natural, alternatively spliced, highly conserved exons containing a premature termination codon that mediates the decay of the transcript spliced, 2) by preventing translation of non-canonical open reading frames (ORFs) or translational inhibitory elements, and by blocking regulatory naturally occurring antisense transcripts (NATs). These techniques together are called targeted augmentation of nuclear gene output (TANGO). Recently, ASOs, targeting PE slide sites of the genes PCCA and SYNGAP1 have been explored. The results demonstrated a significant reduction in PE-containing transcript and doubled the productive mRNA expression. There is another report on targeting PC splicing in SCN1A by ASOs in the animal model for Dravet syndrome wherein the lethality was reduced dramatically (Han et al., 2020).

##### 8.1.4.2 Loss-of-molecular-function variants causing gain-of-function

Here, gene activation occurs due to splice-site loss, premature truncation, or the loss of stop codons. It may be toxic or simply increase the function of the gene. Thus, ASOs can be used to target the toxic allele (White et al., 2015).

##### 8.1.4.3 Gain-of-molecular-function variants resulting in the loss-of-function

Sometimes, the protein becomes nonfunctional due to expansion of repeat or gain of splice sites. ASOs can target these splice sites. For example, studies indicate that in retinitis pigmentosa, CEP290 has been targeted by ASOs (Dulla et al., 2018).

##### 8.1.4.4 Gain-of-molecular-function variants causing gain-of-function

Again, toxic alleles can be targeted by ASOs to treat the toxic gain-of-function resulting from gene duplication. For example, in Amyotrophic Lateral Sclerosis (ALS), the missense allele in superoxide dismutase (SOD) has been targeted by ASOs (Ekhtiari Bidhendi et al., 2018; Wengert et al., 2022).

Most biomedical researchers recognize that ASOs mechanism of silencing the gene expression is similar to RNAi. However, ASOs work in the nucleus influencing transcription and splicing.

### 8.2 Various approaches to patient customization

Large randomized controlled trials (RCTs), the gold standard of clinical research, cannot be used to evaluate ASO treatments for single or very few individuals with a rare disease. Alternatively, these highly individualized n-of-1 clinical trials might be used as an informal “trial of therapy.” Personalized treatment can be viz. 1) Trial therapy for individual treatment, 2) n-of-1 clinical trials, 3) Aggregated n-of-1 trials, and 4) Aggregated n-of-1 case studies (Mirza et al., 2017). For cross-disease treatment, the classical concept of n-of-1 trial design is not appropriate, whereas ASO therapeutics strongly provide the evidence to alter the diseases with the novel, advanced trial designs, such as the pre-post design with a primary treatment in the natural history phase. These trials recognize the importance of integrating patient safety with clinical practice: each trial must be designed to provide generalizable knowledge. Ethical assessment could be customized to the risks and burdens of both activities, as well as the certainties in individual benefit and deviation from standard patient care (Mitchell, 2015). n-of-1 clinical trials typically design to single subjects periodically which receive active treatment or placebo in an ABAB multiple crossover design, ideally in a randomized and blind manner. The aim of ASO therapeutics alter the course of diseases with sustained long term effect which persist after stopping the treatment (Park et al., 2020). Table 4.

**TABLE 4:** Framework of approaches in Patients customization.

Features	Trial of therapy	n-of-1 clinical trials	Aggregated n-of-1 trials	Aggregated n-of-1 case studies
Aim	For individual optimize care	Generalized knowledge individual optimize care	For individual optimize care	Generalized knowledge individual optimize care
Design	Clinical supervision or care	Experimental trial design	Clinical care	Experimental trial design
Study protocol	No	Yes	Yes	Yes
Factor determining the treatment population	Clinical need	Requirements research	Clinical need	Requirements research
Interventions	Individualized (dose, duration, route)	Standardized dose (dose, duration, route)	Individualized (dose, duration, route)	Standardized (dose, duration, route)
Analysis of data	Individual level	Individual level	Aggregated individual level	Aggregated individual level

### 8.3 Applications

ASOs directly exert their effect at the genetic level to halt the production of an undesirable or overabundant protein (Chan et al., 2006). As a result, they have a scope in the clinical application for numerous diseases and disorders. Some of the applications are highlighted below:

• Spinal muscular atrophy (SMA): SMA is a neuromuscular disorder caused by loss of motor neurons in the spinal cord, weakness and atrophy of muscles involved in the movement, and severe disability. The loss of the SMN1 gene results in a significant decrease in the widely expressed survival motor neuron (SMN) protein. Nusinersen, a splice-switching ASO approved by FDA, binds to the intronic splicing suppressor site in SMN2 mRNA intron 7 and promotes SMN2 exon 7 to generate the full-length SMN protein (Haché, et al., 2016; Edinoff, et al., 2021).
• Duchenne Muscular Dystrophy (DMD): DMD is a genetic disorder characterized by progressive muscle wasting, resulting in loss of function in the dystrophin gene. It is a neuromuscular disordered characterized by muscle degeneration and severe cardiomyopathy. The most promising treatment is exon skipping ASO which effectively restores and improves the function of dystrophin in skeletal muscle (Heemskerk et al., 2009; Nguyen and Yokota, 2019).
• Huntington’s Disease (HD): This is a CNS neurodegenerative disorder involving uncontrolled excessive motor movements and cognitive deficits. It occurs due to CAG repeat expansions in exon 1 of the huntingtin gene, resulting in a polyglutamine’s pathogenic expansion. Tominersen is an ASO, presently in clinical trials, that targets the huntingtin gene mRNA and decreases gene expression, hampering protein production (Lane et al., 2018; Hawellek et al., 2022).
• Alzheimer Disease (AD): AD is the most common and devastating neurodegenerative disorder associated with causative proteins tau and amyloid beta. Currently, 2′MOE (2′-O-methoxyethylribose) ASO is under phase I/II to evaluate the safety and target microtubule-associated protein tau (MAPT) mRNA to decrease the tau protein level (Schoch and Miller, 2017).
• Ebola Virus Infection: A recent study showed the locked nucleic modified ASO targeting the host factor Niemann-Pick C1 which is needed by the virus to get into the host cytoplasm. *In vitro* results showed a decrease in the virus titer and 94% knockdown efficiency in the murine cell lines. This result indicates that ASO could be a promising therapeutic for the ebola virus (Javanbakht et al., 2018).
• Hepatitis B infection: The locked nucleic acid (LNA) single-stranded oligonucleotide targets the liver to treat chronic hepatitis B by RNase-H-mediated degradation. To get the liver-specific effect, LNA oligonucleotide is conjugated with three N-acetyl galactosamines by binding to asialoglycoprotein, which particularly expresses in the liver. The antiviral effect of conjugated locked nucleic is higher than that of non-conjugated (Javanbakht et al., 2018).
• SARS-CoV-2 infection: Two types of ASOs have been reported wherein one is conventional modified phosphorothioate and the others are LNA GapmeR that targets the 5′ unsaturated region (5′-UTR), open reading frame 1a and 1b (ORF1a and ORF1b). The most putative GapmeR targets the 5′-UTR in the virus cycle, and five ASO candidates target the ORF1a and ORF1b and 5′-UTR (Neuman et al., 2004). Another study showed that the intranasal administration of locked nucleic acid inhibits replication (Barrey et al., 2020).
• Osteoarthritis: Arthritis involves loss of proteoglycans by A disintegrin and metalloproteinase with thrombospondin motifs (ADAMTS) during cartilage degeneration. The LNA ASO inhibits these ADAMTS and reverses the degeneration in the monolayer culture of human OA chondrocytes. ASO showed a constant discharge profile with hydrogels up to 14 days, identified by flow cytometry and confocal microscopy (Garcia et al., 2019).
• Diabetes: It has been found that second-generation ASO could be used in diabetic-associated retinopathy, which downregulates the multiple signal pathways that are linked with ocular angiogenesis and vascular leakage (Hnik et al., 2009).
• Hereditary transthyretin-mediated amyloidosis: Inotersen is an ASO approved by the FDA for the treatment of transthyretin-mediated amyloidosis. The drug targets the transthyretin (TTR) mRNA (Mathew et al., 2019). Markov et, al 2019, Lindgren et al., 2000.

## 9 Conclusion

The success stories of Fomivirsen, pegaptanib, and patisiran give hope for developing many more ON therapeutics. The major driving force for the growth of OT is the treatment of untreatable diseases and personalized therapy. The global market for oligonucleotide therapeutics synthesis is expected to cross 14.1 billion USD in 2026 from the present USD 6.3 billion (in 2021), growing at a CAGR of 17.6% in this period. Currently, 15 ONs are in the clinic, of which four are used to treat DMD, in addition to several clinical trials going on for neurological, cardiovascular, hepatic, and ophthalmic disorders. ASOs have been developed in treatment of genetic diseases as well as prevention of disease complications. However**,** it also needs to be considered that these drugs are associated with risks, and ethical and clinical issues must be sorted out. Therefore, many challenges and obstacles must be dealt with before successful advancement to clinical applications. The infant stage of ON therapeutics requires continuous efforts and advances to combat the associated issues.
